# Predictive factors for postoperative visual function in eyes with epiretinal membrane

**DOI:** 10.1038/s41598-023-49689-8

**Published:** 2023-12-14

**Authors:** Misa Miyazato, Yume Iwashita, Kazushi Hirono, Jared Ching, Kentaro Nakamura, Tatsuya Inoue, Ryo Asaoka, Yasuo Yanagi, Maiko Maruyama-Inoue, Kazuaki Kadonosono

**Affiliations:** 1 Department of Ophthalmology and Micro-Technology, Yokohama City University, 4-57 Urafune, Minami-ku, Yokohama, Kanagawa 232-0024 Japan; 2 Department of Ophthalmology, Addenbrookes Hospital, Cambridge, UK; 3 Department of Ophthalmology, Seirei Hamamatsu General Hospital, Shizuoka, Japan; 4 Seirei Christopher University, Shizuoka, Japan

**Keywords:** Prognostic markers, Retinal diseases

## Abstract

Our current study aimed to investigate the association of preoperative OCT parameters with visual function after vitrectomy surgery in eyes with epiretinal membrane (ERM). This study enrolled 33 eyes with ERM that underwent vitrectomy surgery. In addition to visual acuity (VA), metamorphopsia was measured pre- and postoperatively for each eye. Using the preoperative horizontal and vertical OCT images, SUKIMA (the gap area between the ERM and retinal surface) was measured respectively and the average of horizontal SUKIMA and vertical SUKIMA was used for the analysis. The associations of baseline parameters (age, axial length, preoperative central retinal thickness [CRT], inner nuclear layer [INL] thickness, ectopic inner foveal layer [EIFL] and SUKIMA) with postoperative VA, the change in VA, postoperative metamorphopsia and the improvement in metamorphopsia were investigated using multivariate regression analysis followed by the model selection. The result suggested that age and INL thickness were related to the postoperative VA, whereas age and preoperative CRT were significantly associated with the change in VA. In contrast, only SUKIMA was correlated with the postoperative metamorphopsia, whilst age, EIFL and SUKIMA were associated with the improvement in metamorphopsia. Measuring SUKIMA might be useful for predicting postoperative metamorphopsia and the improvement in metamorphopsia in ERM eyes.

## Introduction

Epiretinal Membrane (ERM) is one of the most common vitreoretinal diseases in the elderly population. The major symptoms of ERM patients are decreased visual acuity and metamorphopsia^1–3^. With the recent advances in optical coherence tomography (OCT) imaging, several studies have investigated the structure–function relationships in eyes with ERM. For instance, significant associations between metamorphopsia and the inner nuclear layer (INL) thickness, central retinal thickness (CRT), ectopic inner foveal layer (EIFL) and the maximum depth of the retinal fold were previously reported in ERM^4–8^. Furthermore, measurement of these parameters preoperatively may be useful for predicting visual outcomes in eyes with ERM.

We recently identified *SUKIMA*, the area of gap between the ERM and the retinal surface, as a novel OCT parameter that associates with metamorphopsia in eyes with ERM^9^. However, our previous report was cross-sectional and did not investigate the correlation between SUKIMA and postoperative visual function (visual acuity and metamorphopsia). It is possible that SUKIMA might be useful for predicting visual function after ERM surgery in addition to other OCT parameters. Therefore, the current study aimed to investigate the correlation between preoperative OCT parameters and visual function after vitrectomy surgery and to evaluate the usefulness of SUKIMA measurement for predicting the visual outcomes in eyes with ERM.

## Methods

This retrospective study enrolled patients with idiopathic ERM who had undergone vitrectomy surgery at our institution. The exclusion criteria included eyes with (i) other retinal diseases such as age-related macular degeneration or diabetic retinopathy, (ii) a previous history of vitreoretinal surgery. Moreover, eyes with ERM foveoschisis, lamellar macular hole and macular pseudohole were also excluded from this study. The present study was approved by the Ethics Committee of Yokohama City University and written informed consent was obtained from each of the participant. All the procedures were performed according to the tenets of the Declaration of Helsinki.

All the patients underwent comprehensive ophthalmologic examinations, including visual acuity (VA) measurement. Additionally, the degree of metamorphopsia was quantified by M-CHARTS (Inami, Tokyo, Japan), and the axial length (AL) was measured using the IOLMaster 700 (Carl Zeiss Meditec AG, Jena, Germany). The M-CHARTS examination was performed in vertical and horizontal directions and measured as MV and MH, respectively. Then the average of MV and MH scores was calculated as Mave for each eye and used for the statistical analysis. VA and M-CHARTS measurements were performed at the final visit and used as postoperative VA and postoperative Mave, respectively.

### OCT measurement

**Figure 1:**
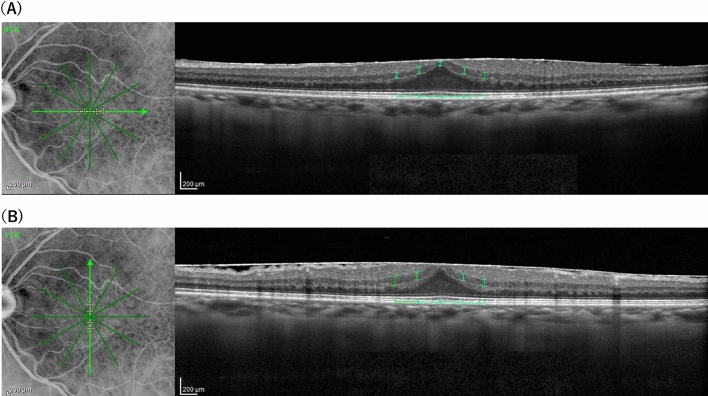
The measurement of the INL thickness in an eye with ERM. A 62-year-old women demonstrated idiopathic ERM in her right eye. Using horizontal (**A**) and vertical (**B**) OCT images, the INL thickness was calculated as the average of 9 points for each eye (fovea, 0.25 mm, 0.50 mm superior, temporal, inferior and nasal from the fovea). ILM: inner nuclear layer, ERM: epiretinal membrane.

**Figure 2:**
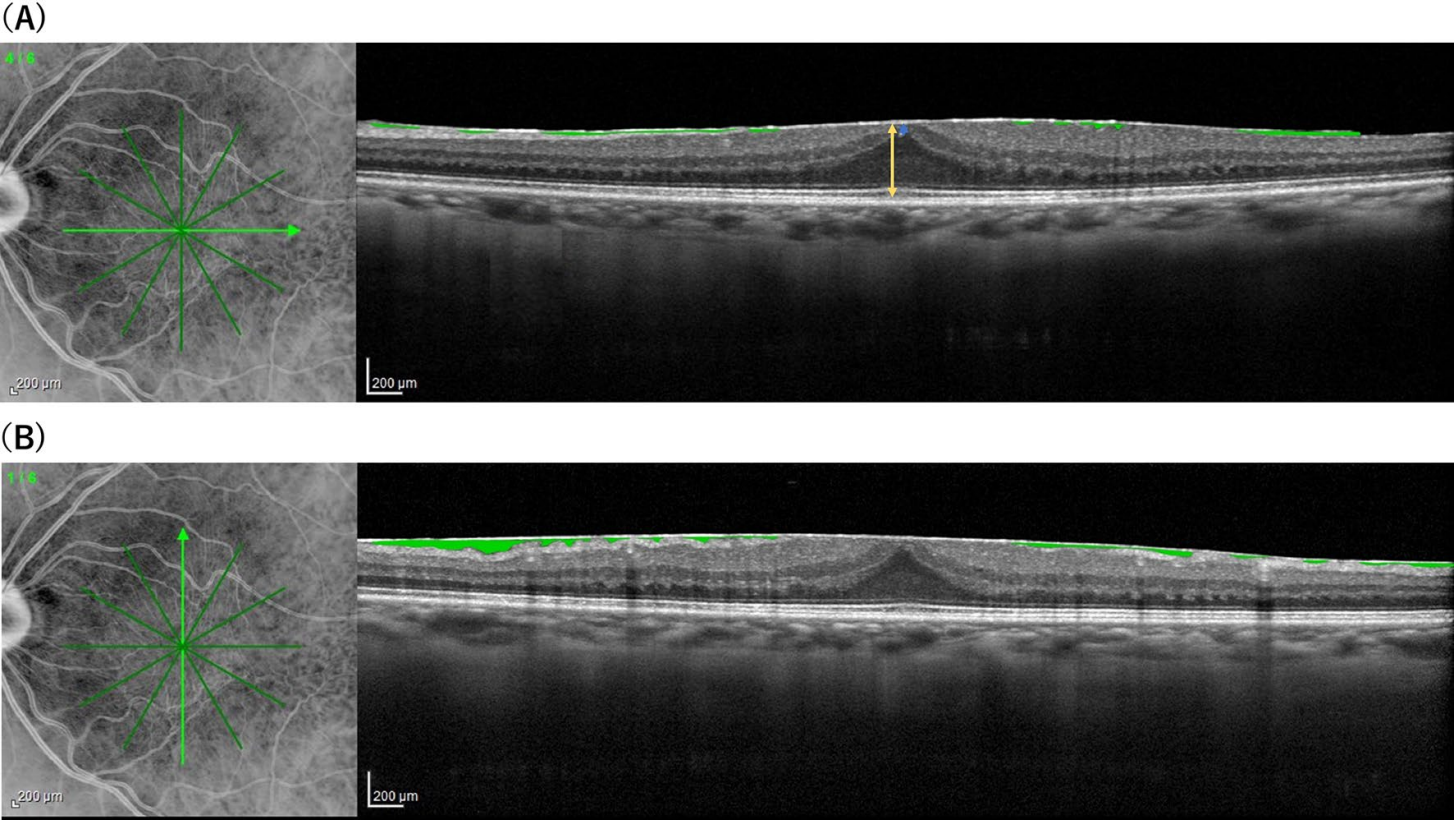
The measurement of SUKIMA, CRT and EIFL in an eye with ERM. (**A**) The CRT (yellow arrow) and EIFL (blue arrow) were measured (422 µm and 22 µm, respectively). In addition, using both horizontal (**A**) and vertical (**B**) OCT scans, SUKIMA was measured with ImageJ software (green area). The average of horizontal and vertical SUKIMA was calculated without magnification correction (0.040 mm^2^) and the value of SUKIMA was corrected to 0.037 mm^2^ using Littmann formula and used for the statistical analysis. CRT: central retinal thickness, EIFL: ectopic inner foveal layer.

We obtained OCT data using the Spectralis OCT (Heidelberg Engineering, Heidelberg, Germany). The preoperative OCT images were used to measure central retinal thickness (CRT), inner nuclear layer (INL) thickness, ectopic inner foveal layer (EIFL) and SUKIMA. In addition to CRT, INL thickness and EIFL were measured as previously reported^7,8^. As shown in Fig. 1A,B, the INL thickness was measured for 9 points (fovea, 0.25 mm, 0.50 mm superior, temporal, inferior and nasal from the fovea). The INL thickness was calculated as the average of 9 points for each eye. The EIFL was defined as continuous hypo- or hyper-reflective band extending from the INL and inner plexiform layer across the foveal region and the thickness was measured between the inner border of the outer nuclear layer and the internal limiting membrane (ILM) at the foveal center (Fig. 2A). SUKIMA was measured in both vertical and horizontal OCT images with scan angle 20° and the average value was used for the analyses (Fig. 2A,B)^9^.

### Magnification correction

Several reports have suggested that the differences of AL affect the lateral magnification in OCT images^10^. To correct ocular magnification in OCT images, we used the Littmann formula, as previously described^11,12^.

$$ t\, = \,p*q*s $$

In this formula, *t* is the actual fundus dimension, factor *p* is the magnification factor for the camera of the imaging system, 3.382 in the Spectralis OCT, *q* is the magnification factor for the individual eye, 0.01306 * (AL [mm] − 1.82), and *s* is the average of horizontal and vertical SUKIMA without magnification correction^13,14^. The value of t was used for the statistical analysis as SUKIMA with magnification correction.

### Surgical technique

All the eyes underwent 25-gauge (25G) pars plana vitrectomy by a single surgeon (KK). Vitrectomy was conducted using Constellation Vision System (Alcon laboratories, Fort Worth, TX). ERM was treated with 25G GRIESHABER MAXGrip Forceps (Alcon Grieshaber, Schaffhausen, Switzerland). After the ERM peeling, indocyanine green dye was used to enhance visualization during ILM peeling. Sulfur-hexafluoride gas or air tamponade was performed at the discretion of the surgeon.

### Statistical analysis

Wilcoxon signed rank test was conducted to compare pre- and postoperative logMAR VA or Mave. Using linear regression analyses, we investigated the relationships (i) between SUKIMA and preoperative logMAR VA, (ii) between SUKIMA and postoperative logMAR VA, and (iii) between SUKIMA and the change in logMAR VA after vitrectomy. Moreover, the correlations (i) between SUKIMA and preoperative Mave, (ii) between SUKIMA and postoperative Mave, and (iii) between SUKIMA and the improvement in Mave were analyzed. Then, multivariate analyses followed by the model selection was performed to identify the optimal model for visual function using the second-order bias-corrected Akaike information criterion (AICc) index. Specifically, among age, AL, preoperative CRT, INL thickness, EIFL and SUKIMA, the optimal models for postoperative logMAR VA, the change in logMAR VA, postoperative Mave and the improvement in Mave were identified, respectively.

The AIC is a common statistical measure in model selection, and the corrected form, AICc, gives an accurate estimation even when the sample size is small^15,16^. The selected variables are regarded as being statistically significant. All statistical analyses were performed using R 3.4.3 (The R Foundation for Statistical Computing, Vienna, Austria).

## Results

The baseline demographic data are shown in Table 1. The present study enrolled 33 eyes of 33 patients (13 males and 20 females) with idiopathic ERM. Of these, 29 eyes underwent vitrectomy combined with phacoemulsification and aspiration (PEA) and intraocular lens (IOL) implantation, and 4 eyes vitrectomy alone. The mean age of the participants was 66.3 ± 10.6 years. The logMAR VA significantly improved from 0.19 ± 0.28 to − 0.005 ± 0.11 (P < 0.001, Wilcoxon signed rank test). In addition, the Mave was significantly improved from 0.61 ± 0.35 to 0.38 ± 0.28 (P < 0.001; Wilcoxon signed rank test).

**Table 1:** Demographic data.

	Preoperative	Postoperative	P value
Number of eyes	33		
Male: female	13:20		
Age (years)	66.3 ± 10.6		
Duration (months)	8.1 ± 6.0		
AL (mm)	24.1 ± 1.6		
SUKIMA (mm^2^)	0.14 ± 0.08		
INL thickness (µm)	74.5 ± 40.4		
EIFL (µm)	60.8 ± 99.8		
CRT (µm)	411 ± 146	363 ± 82	0.007
LogMAR VA	0.19 ± 0.28	− 0.005 ± 0.11	< 0.001
MV	0.68 ± 0.48	0.45 ± 0.40	0.0011
MH	0.55 ± 0.33	0.31 ± 0.27	< 0.001
Mave	0.61 ± 0.35	0.38 ± 0.28	< 0.001

AL: axial length, INL: inner nuclear layer, EIFL: ectopic inner foveal layer, CRT: central retinal thickness, logMAR: logarithm of the minimum angle of resolution, VA: visual acuity, MV: vertical metamorphopsia score, MH: horizontal metamorphopsia score, Mave: average metamorphopsia score.

Univariate analyses suggested that there was no significant correlation between SUKIMA and pre- and postoperative logMAR VA and the change in logMAR VA (p = 0.415, p = 0.57, p = 0.52, respectively, linear regression analysis). Multivariate analysis followed by AICc model selection suggested the optimal model for postoperative logMAR VA included age and the INL thickness. On the other hand, the optimal model for the change in logMAR VA included age and preoperative CRT. The optimal models for the postoperative logMAR VA and the change in logMAR VA were as follows:

$$ \begin{aligned} {\text{Postoperative logMAR VA}} & = - 0.{34}\, + \,0.00{39}\left( { \pm \,0.00{17}} \right) \times {\text{ Age}}\, \hfill \\  & \quad + 0.000{97}\left( { \pm \,0.000{45}} \right) \times {\text{ INL thickness }}({\text{AICc}} =  - {5}0.{7}). \hfill \\ \end{aligned} $$

$$ \begin{aligned} {\text{Change in logMAR VA}}&  = \,0.{64} - 0.00{83}\left( { \pm \,0.00{38}} \right) \times {\text{ Age}} \hfill \\ & \quad - 0.000{7}0\left( { \pm \,0.000{27}} \right)\, \times \,{\text{preoperative CRT }}({\text{AICc}}\, = \,{1}.{2}) \hfill \\ \end{aligned} $$

**Figure 3:**
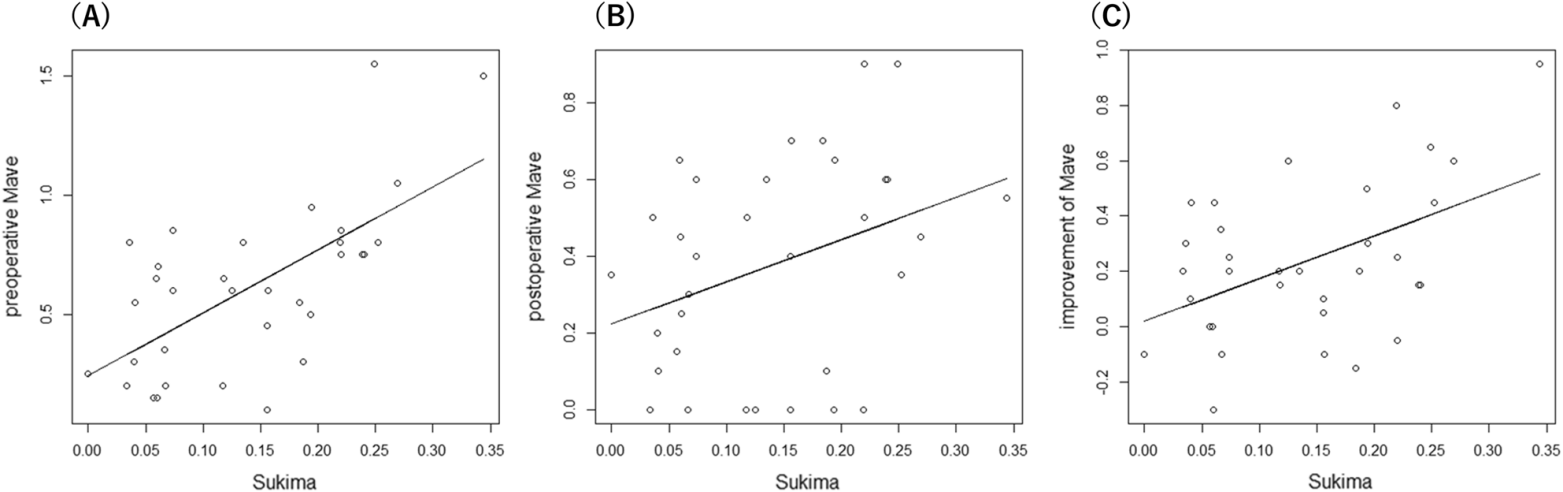
The correlation between SUKIMA and pre- and postoperative Mave and the improvement of Mave. There were significant correlations between SUKIMA and preoperative Mave (**A**, r = 0.613), between SUKIMA and postoperative Mave (**B**, r = 0.296), and between SUKIMA and the improvement of Mave (**C**, r = 0.470). Mave: the average of horizontal and vertical metamorphopsia score.

Univariate analyses suggested that SUKIMA was significantly associated with preoperative Mave (r = 0.613, p = 0.00015) and the improvement of Mave (r = 0.470, P = 0.0058), while a tendency toward significance was observed between SUKIMA and postoperative Mave (r = 0.296, P = 0.094; linear regression analysis, Fig. 3). As a result of the AICc model selection, the optimal model for postoperative Mave included only SUKIMA. Furthermore, the optimal model for the improvement of Mave included age, EIFL and SUKIMA, as follows (Table 2).

$$ {\text{Postoperative Mave}}\, = \,0.{24}\, + \,0.{97}\left( { \pm \,0.{56}} \right) \times {\text{SUKIMA}}({\text{AICc}}\, = \,{11}.{6}). $$

$$ \begin{aligned} {\text{Improvement of Mave}} & = 0.58 - 0.0081\left( { \pm \,0.0039} \right) \times {\text{ Age}} \hfill \\ & \quad - 0.00076\left( { \pm \,0.00041} \right) \times {\text{ EIFL}}\, + \,1.71\left( { \pm \,0.48} \right) \times {\text{SUKIMA}}({\text{AICc}}\, = \,4.2). \hfill \\ \end{aligned} $$

**Table 2:** The optimal model for pre- and postoperative Mave and the improvement of Mave.

Variable	Preoperatove Mave		Postoperative Mave		Improvement of Mave	
	Coefficient	SE	Coefficient	SE	Coefficient	SE
Age	N.S.		N.S.		− 0.0081	0.0039
AL	N.S.		N.S.		N.S.	
Preoperative CRT	N.S.		N.S.		N.S.	
INL thickness	N.S.		N.S.		N.S.	
EIFL	N.S.		N.S.		− 0.00076	0.00041
SUKIMA	2.54	0.59	0.97	0.56	1.71	0.48

Mave: average metamorphopsia score, SE: standard error, N.S.: not selected, AL: axial length, CRT: central retinal thickness, INL: inner nuclear layer, EIFL: ectopic inner foveal layer.

## Discussion

In the present study, we aimed to examine the correlation between SUKIMA and visual function after vitrectomy surgery in eyes with ERM. Consistent with our previous work, SUKIMA with magnification correction was significantly related to preoperative matamorphopsia^9^. We also found that SUKIMA was associated with the postoperative Mave and the improvement of Mave, respectively. Taken together, it is possible that SUKIMA might be a good predictor of not only postoperative metamorphopsia but also the improvement in metamorphopsia.

Our result with the AICc model selection indicated age and INL thickness were significantly related to postoperative logMAR VA and age and preoperative CRT were correlated with the change in logMAR VA but SUKIMA was not. Our previous report suggested SUKIMA was related to VA in addition to metamorphopsia^9^. This discrepant result seems to be due to the difference in patients’ background or the influence of cataract surgery. In our current study, 29 out of 33 eyes had undergone vitrectomy combined with PEA + IOL and there is a possibility that the removal of cataract might contribute largely to the improvement in VA. However, the cataract grading was not taken into consideration, and this is one of the limitations in the present study.

Interestingly, our current study indicated SUKIMA was associated with pre- and postoperative metamorphopsia, and at the same time, with the improvement of metamorphopsia. Thus, ERM patients with larger SUKIMA suffer from worse distortion pre- and postoperatively but benefit from greater overall improvement in metamorphopsia. The association between SUKIMA and postoperative metamorphopsia might be directly influenced by the association between SUKIMA and preoperative metamorphopsia. Indeed, Pearson’s correlation coefficient was 0.613 between SUKIMA and preoperative Mave, and 0.296 between SUKIMA and postoperative Mave. Moreover, it is possible that greater improvement in metamorphopsia in eyes with larger SUKIMA might be associated with surgical difficulty. Kim et al. previously indicted a significant correlation between the difficulty of ERM removal and the extent of ERM-retinal adhesion in eyes with idiopathic ERM^17^. They speculated that the tissue-free space between the ERM and retinal surface may facilitate ERM peeling during vitrectomy surgery compared to ERM-retinal adhesion. Further studies would be needed to clarify the association between SUKIMA and surgical difficulty, however ERM with larger SUKIMA might be peeled more easily.

The present study included several limitations. Firstly, this study was retrospective in nature and the number of enrolled eyes was relatively small. Secondly, SUKIMA was measured with all scan length (20°, 5.8 mm) using the vertical and horizontal OCT images and the ILM thickness and EIFL were measured as previously described, therefore the range of measurement is different between SUKIMA and other OCT parameters. The difference in the measurement range might influence our result. In addition, the period of follow up was variable and relatively short in the present study. Kinoshita et al. reported horizontal and vertical metamorphopsia score improved up to 6 and 12 months after vitrectomy surgery in eyes with ERM^18^. Further investigation with longer follow-up is warranted in the future.

In summary, SUKIMA, a gap between the ERM and retinal surface, is a good predictor for postoperative metamorphopsia after vitrectomy surgery. Furthermore, greater improvement of metamorphopsia could be expected in ERM with larger SUKIMA, suggesting the possibility that the presence of SUKIMA facilitates ERM peeling during vitrectomy surgery.

## Data Availability

The datasets used and analysed during the current study are available from the corresponding author on reasonable request.
